# Interleukin-10 Inhibits Bone Resorption: A Potential Therapeutic Strategy in Periodontitis and Other Bone Loss Diseases

**DOI:** 10.1155/2014/284836

**Published:** 2014-02-16

**Authors:** Qian Zhang, Bin Chen, Fuhua Yan, Jianbin Guo, Xiaofeng Zhu, Shouzhi Ma, Wenrong Yang

**Affiliations:** ^1^Institute and Hospital of Stomatology, Nanjing University Medical School, 30 Zhongyang Road, Nanjing, Jiangsu 210008, China; ^2^School and Hospital of Stomatology, Fujian Medical University, 246 Yangqiaozhong Road, Fuzhou, Fujian 350002, China; ^3^School of Life and Environmental Science, Waurn Ponds Campus, Deakin University, 75 Pigdons Road, Geelong, VIC 3216, Australia

## Abstract

Periodontitis and other bone loss diseases, decreasing bone volume and strength, have a significant impact on millions of people with the risk of tooth loss and bone fracture. The integrity and strength of bone are maintained through the balance between bone resorption and bone formation by osteoclasts and osteoblasts, respectively, so the loss of bone results from the disruption of such balance due to increased resorption or/and decreased formation of bone. The goal of therapies for diseases of bone loss is to reduce bone loss, improve bone formation, and then keep healthy bone density. Current therapies have mostly relied on long-term medication, exercise, anti-inflammatory therapies, and changing of the life style. However there are some limitations for some patients in the effective treatments for bone loss diseases because of the complexity of bone loss. Interleukin-10 (IL-10) is a potent anti-inflammatory cytokine, and recent studies have indicated that IL-10 can contribute to the maintenance of bone mass through inhibition of osteoclastic bone resorption and regulation of osteoblastic bone formation. This paper will provide a brief overview of the role of IL-10 in bone loss diseases and discuss the possibility of IL-10 adoption in therapy of bone loss diseases therapy.

## 1. Introduction

Bone remodeling is a dynamic lifetime process through the resorption of old bone by osteoclasts and the subsequent synthesis of new bone by osteoblasts. These two closely coupled events are responsible for renewing the skeleton while maintaining its anatomical and structural integrity. Under normal conditions, the amount of absorbed bone equals the regenerated bone; bone remodeling proceeds in recycles in which osteoclasts adhere to bone and subsequently remove it by acidification and proteolytic digestion. Once the osteoclasts leave the resorption sites, osteoblasts will migrate to the resorption sites and start forming new bone by secreting osteoid, which is eventually mineralized [1, 2].

Osteoclasts are specialized cells derived from haematopoietic lineage such as monocytes/macrophage. They develop and adhere to bone matrix and then secrete acid and lytic enzymes to degrade bone [3, 4]. The increase in number and/or activity of bone-resorbing osteoclasts could contribute to excessive bone resorption, disrupts the balance between bone resorption and bone formation, and results in the loss of bone. This may consequently lead to the following bone loss diseases: osteoporosis, rheumatoid arthritis, periodontal bone absorption, malignancy-related skeletal diseases, and so on [5–8].

Periodontitis, a bone loss disease, is a chronic inflammatory disease. As for periodontitis, destruction of connective tissue and alveolar bone can occur and finally results in the loss of teeth. Therefore, it is very critical for periodontists if we can develop a method to inhibit bone resorption and promote alveolar bone regeneration. Given the role of IL-10 in bone remodeling, the use of IL-10 for inhibiting bone resorption and reducing inflammation may be beneficial for the treatments of periodontitis.

## 2. Cytokines in Bone Metabolism

It is well known that bone loss diseases are affected by inflammation, hereditary factors, hormones, aging, life style, and so on. Hence, it is imperative for us to develop other therapeutic methods to improve clinical outcome. For decades, a number of attempts have been made to achieve good prognosis for these diseases [9–11]. In particular, much attention has been paid to the importance of cytokines in the pathogenesis of bone resorption. The existing evidences suggested that various cytokines play an important role in both physiologic and pathologic bone resorption [12–14]. Numerous of cytokines exists in the bone tissue, and their functions in bone formation and resorption are still not well understood. It has been demonstrated that receptors in the proinflammatory cytokines IL-1, IL-6, and TNF-*α* are present on osteoclast precursor cells and mature osteoclasts [13, 15]. A breakthrough in understanding the mechanism is that cytokines regulate proliferation and differentiation of mononuclear preosteoclasts into osteoclast progenitors and fusion of the preosteoclasts into multinucleated osteoclasts [16, 17]. Zhao and coworkers showed that the receptor activator of nuclear factor *κ*B ligand (RANKL), which was secreted by live osteocytes, promotes osteoclastogenesis [18]. Moreover, osteoprotegerin (OPG), as a soluble decoy receptor for RANKL, is also a crucial regulator of osteoclastogenesis [14, 19–22]. OPG can block osteoclastogenesis and maintain normal bone mass by binding RANKL and blocking interaction with RANK. Furthermore, *in vitro* and *in vivo* studies have shown that many cytokines elaborated by inflammation, tumor necrosis factor *α* (TNF-*α*), and IL-1 may be attributed to osteoclast differentiation and activation by regulating the production of RANKL and/or OPG [4, 7, 23].

In addition, TNF-*α* and IL-1 promote resorption activity of osteoclasts by increasing macrophage colony stimulating factors (M-CSF). M-CSF can bind to its receptor, c-fms, on precursor cell for differentiation via the actions of RANKL [24].

## 3. IL-10 and Bone Metabolism

IL-10 is a potent anti-inflammatory cytokine that suppresses both immunoproliferative and inflammatory responses. So understanding of the role of IL-10 in the bone loss diseases is essential. IL-10 was first identified at molecular level by the DNAX Research Institute [25]. As a factor produced by T helper 2 (Th2) cells, IL-10 inhibits the production of cytokines by Th1 cells [26]. IL-10 has been subsequently shown additional stimulative effects on thymocytes, B cells, and mast cells. It is well known that IL-10 is actually produced by many other cell types, including B cells, mast cells, eosinophils, macrophages, and dendritic cells (DCs), and a large number of subsets of T cells such as CD8^+^ T cells and antigen-driven regulatory CD4^+^ T cells [27] in mouse and human systems.

IL-10 can downregulate the synthesis of proinflammatory cytokines and chemokines, such as IL-1, IL-6, and TNF-*α* [28, 29]. It can also downregulate the synthesis of nitric oxide, gelatinase, and collagenase. Specific neutralization of IL-10 results in upregulating the synthesis of IL-1 and TNF-*α* [30, 31]. Therefore, IL-10 has been also regarded as an important regulator of bone homeostasis, in homeostatic and inflammatory conditions [32–34].

Recent studies have indicated that the polymorphisms of IL-10 gene, which may affect IL-10 production, are associated with reduced bone mineral density (BMD) in postmenopausal women who were prone to suffered from osteoporosis [35, 36]. However, serum levels of IL-10 are significantly lower in the postmenopausal osteoporotic patient than in postmenopausal healthy women [37]. The low level of IL-10 results in the insufficient inhibition of the proinflammatory cytokines and collagenase, which may have an impact on osteoporosis development [38]. Animal studies have confirmed that lack of IL-10 leads to femur bone loss [39, 40] and alveolar bone loss [33, 40, 41], providing further evidence of bone metabolism by IL-10 [39]. In the oral bone lytic diseases, such as periodontitis and periapical lesions, IL-10 has been shown as an important regulator of alveolar bone homeostasis [40, 42–44]. Herein, we speculate that IL-10 acts on the bone loss diseases based on the following criteria.

*(1) IL-10 Inhibits Osteoclasts Formation. *Xu and coworkers showed that IL-10 had potent inhibitory effects on osteoclastogenesis in the 1990s [45]. Others suggested IL-10 could directly inhibit osteoclast formation [46, 47]. The inhibitory effect of IL-10 on osteoclast formation through a direct action on osteoclast precursors was reported [47, 48]. Moreover, enhanced osteoclastogenesis has been observed in cultures of bone marrow macrophages deficient in IL-10 production [49]. The molecular mechanism of this inhibition indicated that IL-10 upregulated osteoprotegerin (OPG) expression but downregulated expression of the receptor activator of NF-*κ*B ligand (RANKL) and colony-stimulating factor-1 (CSF-1) [50]. *In vitro* test showed that IL-10 may inhibit osteoclastogenesis by reducing nuclear factor of activated T cells (NFAT) c1 expression [46, 51].

Bone resorption was mediated largely by local production of proinflammatory cytokines, such as TNF-*α* and IL-1 [4, 17, 23, 25, 26]. These cytokines may act by directly enhancing proliferation and activity of cells in the osteoclast lineage or by indirectly affecting the production of osteoclast differentiation factors such as RANKL and OPG via osteoblast/stromal cells [4]. IL-10 has been recognized to have potent anti-inflammatory activity for a long time, and it is demonstrated to be an important endogenous suppressor of infection-stimulated bone resorption *in vivo* [52]. In conclusion, IL-10 suppresses osteoclastic differentiation via the above several aspects.

*(2) IL-10 Promotes Osteoblastic Differentiation Overall.* van Vlasselaer and coworkers suggested that IL-10 downregulated early steps of osteogenic differentiation in murine bone marrow cells through the inhibition of transforming growth factor-beta 1 (TGF-*β*1) production [53, 54]. However, Dresner-Pollak and coworkers indicated that reduced generation of osteoblasts has been found in bone marrow cell cultures obtained from IL-10(−/−) mice, and IL-10(−/−) mice develop the hallmarks of osteoporosis, that is, decreased bone mass, increased mechanical fragility, and suppressed bone formation [39]. They also suggested that cytokines and inflammatory mediators, including TNF-*α*, IFN-*γ*, and NO, which are known to be upregulated in the IL-10(−/−) mice, had deleterious effects on the differentiation, proliferation, and function of osteoblasts [39]. The inhibitory effects in early stages of osteogenic differentiation might be neutralized eventually by the downregulation of inflammatory cytokines of infection, such as TNF-*α*. In other words, IL-10 enhances the osteoblastic differentiation eventually.

## 4. IL-10 in Periodontal Diseases and Periodontal Treatment

As an important anti-inflammatory cytokine, IL-10 plays a vital role in periodontal diseases. The lockout of IL-10 may result in accelerating alveolar bone absorption and decreasing bone formation [38–42]. Meanwhile, animal models by the IL-10 knockout mice demonstrated that the IL-10 had the anti-inflammation effect in periodontitis [43]. Currently some studies revealed that polymorphisms of IL-10 gene promoter were involved in the development of periodontal diseases. The specific genotypes (−819TT/−592AA) with low IL-10 expression may aggravate the inflammation response and cause the overgrowth of gingival [55]. The haplotype ATA of IL-10, as a “low interleukin-10 producer,” was proved as a risk indicator for generalized aggressive periodontitis [56]. Moreover, others observed that these single-nucleotide polymorphisms (SNPs) of IL-10, including −1082(−1087)A/G, −819(−824)C/T and −592(−597)C/A, were associated with the generalized chronic periodontitis and/or aggressive periodontitis [57–60], which elucidates the role of IL-10 in periodontal diseases.

Smoking is well recognized as a risk factor of periodontitis, and it has been reported that smokers are several times more likely to suffer from periodontitis than nonsmokers [61]. Ebersole and coworkers indicated that smoking may cause the remarkable Th2 response and the elevating of IL-10 [62, 63], suggesting that the periodontitis could be worse by smoking.

Applications of IL-10 have been explored by several animal models [38–41, 64–66]; further evidences are still needed in the future study.

## 5. Future Perspective

Diseases of the bone loss are severely influencing quality of people's health and life. As IL-10 can contribute to the maintenance of bone mass by inhibition of osteoclastic bone resorption and stimulation of osteoblastic bone formation, we hypothesize that utility of IL-10 will be a novel therapeutic strategy in bone loss diseases. It might especially be an attractive treatment option for inflammation-related bone loss, such as periodontal diseases, periapical lesions, and inflammatory bowel disease. The use of IL-10 will be helpful to accelerate the healing process of fracture and enhance osseointegration with dental implantation in osteoporotic subjects.

## 6. Conclusions

The strategy of inhibiting osteoclastogenesis and enhancing osteoblastic differentiation can be beneficial for the treatment of bone loss diseases. Given the fact that IL-10 can inhibit bone loss, the use of IL-10 will be an effective therapeutic method for periodontitis and other bone loss diseases. We expect that IL-10 protein therapy could be tested by examining bone loss in ligature-induced periodontitis and bone turnover in ovariectomized rats. The strategies presented in this review can facilitate the future research on IL-10 in bone loss diseases.
